# Shared decision making in patients with severe mental disorders

**DOI:** 10.1192/j.eurpsy.2023.86

**Published:** 2023-07-19

**Authors:** J. Hamann

**Affiliations:** Bezirksklinikum Mainkofen, Deggendorf, Germany

## Abstract

**Abstract:**

Shared decision making (SDM) has found its way into mental health care to a limited extent only, and especially “challenging” patients do not benefit from this approach. In this lecture we will describe barriers to shared decision making among mental health professionals and among patients. Integrative approaches will be presented that meet the needs of patients and mental health staff when aiming at implementing SDM in acute mental health settings. Finally, best practice examples will illustrate that SDM actually can be implemented in the very acute settings/treatment phases and yields positive results.

**Disclosure of Interest:**

None Declared

